# Predictors, Complications, and Clinical Outcomes of Cerebrospinal Fluid Leak Post Endoscopic Endonasal Skull Base Surgery

**DOI:** 10.3390/brainsci16010019

**Published:** 2025-12-24

**Authors:** Alejandro Vargas-Moreno, Sami Khairy, Mouaz Saymeh, Damanpreet Kaur Lang, Sara K. Dabbour, Jessica Rabski, Shaun Kilty, Fahad Alkherayf

**Affiliations:** 1 Division of Neurosurgery, Department of Surgery, The Ottawa Hospital, Ottawa, ON K1Y 4E9, Canadaskhairy@toh.ca (S.K.); jrabski@toh.ca (J.R.); falkherayf@toh.ca (F.A.); 2 The Ottawa Hospital Research Institute, The Ottawa Hospital, Civic Campus, 1053 Carling Ave., Ottawa, ON K1Y 4E9, Canada; mosaymeh@ohri.ca; 3 College of Medicine, Alfaisal University, Riyadh 11533, Saudi Arabia; 4 Department of Otolaryngology—Head and Neck Surgery, University of Ottawa, Ottawa, ON K1Y 1J8, Canada; skilty@toh.ca; 5 Division of Neurosurgery, University of Ottawa, Ottawa, ON K1N 6N5, Canada

**Keywords:** CSF leak, endoscopic endonasal surgery, pedicled vascularized flap, intraoperative monitoring, skull base tumor

## Abstract

Background: Postoperative cerebrospinal fluid (CSF) leakage remains a significant complication following endoscopic endonasal skull base surgery (EES), leading to increased morbidity. This study aimed to identify factors and interventions predicting postoperative CSF leaks after EES for intradural skull base tumors and their clinical outcomes. Methods: We retrospectively reviewed data from 542 patients who underwent EES for intradural skull base pathology at the Ottawa Hospital between October 2001 and October 2023. Patient demographics, pre-operative, intraoperative (including reconstruction type), postoperative data, and patient outcomes were collected. Results: A total of 40 patients (7.4%) developed a postoperative CSF leak. The highest rate was in patients with suprasellar lesions (5.9%), followed by anterior cranial fossa lesions (1.1%). Significant predictors included a higher mean Body Mass Index (BMI) (30.4 vs. 26.1, *p* = 0.001). The use of a nasoseptal flap for reconstruction was associated with a significantly lower incidence of CSF leaks (*p* = 0.001). Tumor location, approach type, and dural sealants were not independent factors for the development of CSF leaks. Patients with CSF leaks had significantly longer lengths of stay (16.7 vs. 9.21 days, *p* < 0.001), higher 30-day readmission rates (*p* < 0.001), and increased postoperative sepsis (*p* = 0.021) and diabetes insipidus (*p* < 0.001). Conclusion: This retrospective study shows that higher preoperative BMI is associated with a significant risk of postoperative CSF leaks after EES. Conversely, using a pedicled vascularized flap reduces the risk. Postoperative CSF leaks are linked to increased morbidity, including diabetes insipidus and sepsis, prolonged hospitalization, and higher readmission rates.

## 1. Introduction

Endoscopic endonasal surgery (EES) has become a cornerstone in the surgical treatment of skull-based pathology. The evolution of this approach has enabled the resection of large skull base tumors using minimally invasive techniques. However, postoperative cerebrospinal fluid (CSF) leakage remains one of the most significant complications associated with EES. The presence of CSF leaks can lead to severe consequences, including meningitis, increased morbidity, higher healthcare costs, and prolonged recovery times [1]. Identifying the frequency of these leaks and understanding the associated risk factors are essential for improving patient outcomes [2]. Despite the significance of this issue, data on the risk factors associated with postoperative CSF leaks remain limited, with both patient-specific factors and perioperative interventions likely contributing to this complication [3,4,5].

Previous studies have identified potential risk factors for CSF leaks after EES, including tumor size, body mass index (BMI), previous surgeries, reconstruction techniques, and the use of adjuvants such as lumbar drains [6,7]. However, few reports have compared postoperative CSF leakage rates across different tumor types, locations, sizes, or the need for subarachnoid dissection, factors that influence the skull base repair [8].

In this study, we present our data on endonasal intradural skull base tumor resections. We aimed to identify whether patient-specific factors (patients’ comorbidities, tumor pathology, and tumor location) and perioperative interventions (such as lumbar drain use and type of reconstruction) are independent risk factors for the development of postoperative CSF leaks after an endoscopic endonasal approach for the resection of intradural skull base tumors.

## 2. Methods

The Ottawa Hospital Civic Campus, Ottawa, ON, Canada skull base database was queried to identify cases of endoscopic endonasal skull base surgery for the resection of intradural pathology between October 2001 and October 2023. Records of all adult patients with intradural involvement were screened for inclusion. Lumbar drains (LDs) were used throughout most of the study period for any extended endonasal approaches, and the nasoseptal flap was similarly utilized for reconstruction. LDs were reserved for cases deemed as high risk (larger tumors, patients requiring an extended approach, subarachnoid or parenchymal extension of the mass, or ventricular extension of the lesion). Exclusion criteria included the inability to confirm intradural tumor involvement, incomplete clinical records, and patients with a history of previous CSF leaks, skull base trauma, or active infection at the time of surgery. After the surgical procedure, all patients received a three-day prophylactic antibiotic course with a first-generation cephalosporin.

All patient medical records and operative notes were reviewed in detail. Data collected included the following: Body Mass Index (BMI), ASA class, type of approach, surgical time, blood loss, length of stay, use of lumbar drain, use of dural sealants, use of nasoseptal flap, CSF leak presence, readmissions at 30 and 90 days, tumor pathology, and medical complications (including diabetes insipidus, sepsis, embolic and cardiac complications). Surgical approaches were categorized based on tumor location (anterior cranial fossa, sellar, suprasellar, and posterior cranial fossa). Rates of postoperative CSF leakage for each variable were analyzed using the chi-square test. Comparisons were made between patients with and without CSF leaks.

In cases of CSF leak, the patients were initially managed with a lumbar drain insertion. If non-responsive, re-intervention and revision via endoscopic endonasal approach were performed.

Descriptive statistics are presented as mean, standard deviation, and range for continuous variables and as frequency and percentage for categorical variables. The Shapiro–Wilk test was applied to ensure normal distribution of the data. The chi-squared test, along with Fisher’s exact test, was used to compare categorical variables and determine relationships between them, with a 95% significance level. Statistical analysis was performed using IBM SPSS Statistics version 26.0 software.

## 3. Results

A total of 542 patients with confirmed intradural pathology were included in the final analysis. Of these, 84.1% were classified as ASA classes I–III. The mean operative time was 216 min (range: 17–796), the mean blood loss was 349 mL (range: 100–3500), and the mean length of stay was 9.76 days (Table 1).

Out of the 542 patients, 40 developed CSF leak (7.4%). The highest rate of CSF leak was observed in patients with sellar or suprasellar lesions (5.9%), followed by those with anterior cranial fossa lesions (1.1%), and posterior cranial fossa lesions (0.4%) (Table 2).

Patients who developed a CSF leak had a significantly longer length of stay (*p* < 0.001). Additionally, the presence of a CSF leak was associated with a higher rate of 30-day readmission (*p* < 0.001), as well as the development of sepsis (*p* = 0.021).

Mean BMI was higher in patients developing CSF leaks (30.4 vs. 26.1, *p* = 0.001). There was no statistical difference between ASA classes and the presence of CSF leaks (*p* = 0.83). Tumor location, type of approach (*p* = 0.578), and the use of dural sealants (*p* = 0.195) were not independent factors for the development of CSF leaks.

Eighty-one patients had a lumbar drain placed. According to the approach, 10.86% of the transclival, 40.32% of the anterior cranial fossa, and 11.75% of the transsellar cases received this device. In this high-risk population, the use of lumbar drains was associated with a higher incidence of cases of CSF leaks (*p* = 0.021). In patients in whom a lumbar drain was placed, tumors > 3 cm in size, ventricular or paraventricular extension, or subarachnoid extension of the mass were common findings.

Rates of CSF leakage varied based on tumor pathology. Histological diagnosis was associated with the presence of a CSF leak in patients with non-functioning pituitary adenoma (NFPA) (*p* = 0.019) and with skull base adenocarcinomas (*p* < 0.001) (Table 3).

The documentation of intraoperative CSF leak was associated with post-operative CSF leak development (*p* = 0.001), while the use of a nasoseptal flap for reconstruction was associated with a lower incidence of this complication (*p* = 0.001).

## 4. Discussion

Our clinical cohort exhibited a total CSF leak occurrence rate of 7.4%, which aligns with currently reported and accepted rates in the medical literature [2,4,9]. Based on our data, we conclude that a higher BMI, intraoperative CSF leaks, high-risk cases requiring lumbar drain use, and certain histological types are associated with a higher incidence of this complication.

Increased ICP in overweight and obese patients may place additional strain on surgical reconstructions of the skull base, potentially increasing the risk of postoperative CSF leaks [10]. The pathophysiology behind this association remains hypothetical, but it can be speculated that a mechanism similar to the one linking elevated BMI and spontaneous CSF leaks is at play [11], even in the absence of documented idiopathic intracranial hypertension. In our population, obese patients were more prevalent in the CSF leak group, and this association was statistically significant.

Lumbar drains can be used to prevent intraoperative leaks resulting from significant dural defects caused by tumor resection or surgical access to the tumor. Up to 67% of surveyed American rhinologists routinely use LDs in the management of CSF leaks [12]. However, their indications are not clearly defined, and their use varies considerably among surgeons and centers. Several analyses conclude that there is insufficient evidence to demonstrate that LDs significantly influence postoperative CSF leak recurrence rates following endoscopic CSF leak repair [13].

In our study population, lumbar drain placement was associated with higher rates of postoperative CSF leakage. This association is likely a reflection of selection bias, as lumbar drains are typically placed in patients deemed intraoperatively to be at high risk for developing a postoperative CSF leak—a trend also reported by other groups [7,14,15], reflecting a higher complexity of the case more than a true risk factor. We reserve lumbar drain placement for high-risk cases requiring extended approaches, such as extensive subarachnoid or parenchymal dissection, in which these devices are used to prevent this complication in the postoperative period.

Consistent with the current literature, in our study group, the use of a vascularized flap for skull base reconstruction was associated with a significant reduction in postoperative CSF leakage rates. The greatest advantage of this flap is that it can be harvested endoscopically before creating the skull base defect, eliminating the need for external flap harvesting. Furthermore, it provides the same benefits as other vascularized flaps, namely faster healing and a lower incidence of graft migration. Another major advantage is its ability to cover a very large surface area [16,17].

When comparing tumor locations in this population, patients with sellar or suprasellar lesions exhibited the highest frequency of CSF leak development. Factors contributing to this may include the technical complexity of resection and reconstruction, combined with direct communication with the arachnoid cisterns and ventricular cavities [18]. This contrasts with tumor resection in the anterior fossa, where lower CSF pressure and frontal lobe descent into the surgical defect reduce the risk of leakage [19].

Tumor subgroup analysis revealed that patients with NFPA and adenocarcinomas were more prone to developing CSF leaks. The larger size of these lesions at the time of diagnosis, cisternal extension, and extensive skull base destruction in malignant cases may explain this finding. These factors highlight the importance of identifying high-risk cases to tailor the surgical approach and reconstruction strategy accordingly [20]. Additional studies with a larger population sample in each tumor subgroup would allow us to validate these findings.

Additionally, CSF leak was associated with a higher risk of sepsis as well as other medical complications, such as diabetes insipidus (DI). Postoperative infections following endonasal approaches for intrasellar lesions, including bacterial meningitis, tend to have a low incidence of 1–10%. Risk factors for infection have been described in association with complex tumors, the presence of an external ventricular drain or shunt, and postoperative CSF leaks [21,22]. Although present in our series, the morbidity of these cases was low, and no mortality was associated with these complications.

Several groups have confirmed the association between CSF leaks and DI incidence, with the likelihood of DI increasing with the severity of the CSF leak. A possible mechanism underlying postoperative DI is direct or indirect damage to the posterior lobe or pituitary stalk due to diaphragm destruction, the extent of which is reflected in postoperative CSF leak rates [23]. Surgeons should be aware of the risk of postoperative DI when dissecting the diaphragm and tumor and should aim to minimize damage to the pituitary stalk whenever surgically feasible.

DI was significantly associated with patients in whom CSF leakage was documented in our cohort. As the majority of our cases were sellar and suprasellar, and due to extensive dissection, leading to arachnoid manipulation, this could also contribute to pituitary stalk disruption, explaining this association. It is imperative that this population receives optimal management in order to decrease the added morbidity of these complications.

Analysis of outcomes in patients with postoperative CSF leaks revealed a direct correlation between this complication and both length of stay (LOS) and readmission rates. As healthcare utilization gains increasing attention, addressing factors that prolong LOS becomes crucial. Identifying patients at higher risk for developing this complication is essential, as it could help reduce costs and improve outcomes [24].

Limitations of our study include its retrospective nature and the inherent biases associated with such studies. In addition to this, the analysis relied solely on univariate statistics, which prevented the identification of independent predictors. Factors such as CSF leak flow rate, surgical defect size, and history of previous radiation treatment were not accounted for in our analysis. The inclusion of a wide range of surgical approaches, tumor types, and reconstruction techniques reduces the statistical power of each patient subset and the strength of potential correlations. Finally, reconstruction techniques and case complexity evolved over the course of the study, introducing a variable that cannot be accurately measured and may have impacted the overall results with a temporal bias.

## 5. Conclusions

Higher preoperative BMI and intraoperative CSF were associated with an increased risk of postoperative CSF leaks. Use of a pedicled vascularized nasoseptal flap may be associated with a reduced risk of complications. Patients with postoperative CSF leaks had a higher incidence of diabetes insipidus, prolonged lengths of stay, sepsis, and increased readmission rates. These findings underscore the importance of vigilant intraoperative monitoring and the potential need for targeted strategies to mitigate the risks of CSF leaks in this patient population.

## Figures and Tables

**Table 1:** Demographics and general characteristics of the study population.

Patient Demographics and General Characteristics		
Variable		
ASA Class, no (%)	I	2 (0.4%)
	II	54 (10.2%)
	III	388 (73.5%)
	IV	84 (15.9%)
Approach/Location	Sellar/Suprasellar, no. (%)	434 (80.1%)
	Anterior cranial fossa, no. (%)	62 (11.4%)
	Posterior cranial fossa, no. (%)	46 (8.5%)
Blood loss (Mean in ml-min/max)		340 (100–3500)
LOS (Mean in days)		9.76

LOS: Length of stay.

**Table 2:** Predictors of CSF leak in the study population.

CSF Leak Predictors				
Variable		No CSF Leak (*n* = 502)	CSF Leak (*n* = 40)	*p* Value
ASA Class				0.83
	I	2	0	
	II	51	3	
	III	357	32	
	IV	79	5	
Mean BMI in kg/m^2^		26.1	30.4	0.001
Mean LOS in days		9.21	16.7	<0.001
Lumbar drain, no. (%)				0.021
	Anterior cranial fossa, 25 (40.32%)	37	25	
	Sellar suprasellar, 51 (11.75%)	383	51	
	Transclival, 5 (10.86%)	41	5	
Intraoperative CSF leak, no.		234	40	0.001
Approach/Location				0.578
	Sellar/Suprasellar, no.	402	32	
	Anterior cranial fossa, no.	56	6	
	Posterior cranial fossa, no.	44	2	
Nasoseptal flap use		468	30	0.001
Dural sealant, no (%)		286 (52.8%)	27 (5%)	0.195
Readmission at 30 days, no.		13	31	<0.001
PostOp. Sepsis, no.		1	1	0.021
PostOp. DI, no.		12 (2.2%)	60 (11.1%)	<0.001

DI: Diabetes insipidus.

**Table 3:** CSF leak incidence based on tumor pathology.

Tumor Pathology and CSF Leak					
Variable		Cases	No CSF Leak	CSF Leak	*p* Value
Tumor Histology	NFPA	247	232	15	0.019
	Craniopharyngioma	35	29	6	0.07
	FPA	79	74	5	0.37
	Meningioma	40	35	5	0.4
	Rathke’s cyst	19	16	3	0.28
	Chordoma	17	15	2	0.67
	Adenocarcinoma	3	1	2	<0.001
	Epidermoid cyst	4	3	1	0.25
	Pituitary metastases	5	4	1	0.38

FPA: Functioning pituitary adenoma. NFPA: Non-functioning pituitary adenoma.

## Data Availability

The data associated with the paper are not publicly available but are available from the corresponding author on a reasonable request. Data are in a controlled data storage environment at the Ottawa Hospital.

## References

[1] Lobatto D., Vries F., Najafabadi A., Pereira A.M., Peul W.C., Vlieland T.P.M.V., Biermasz N.R., van Furth W.R. (2018). Preoperative risk factors for postoperative complications in endoscopic pituitary surgery: A systematic review. Pituitary.

[2] Kim J.S., Hong S.D. (2021). Risk factors for postoperative CSF leakage after endonasal endoscopic skull base surgery: A meta-analysis and systematic review. Rhinology.

[3] Fraser S., Gardner P.A., Koutourousiou M., Kubik M., Fernandez-Miranda J.C., Snyderman C.H., Wang E.W. (2018). Risk factors associated with postoperative cerebrospinal fluid leak after endoscopic endonasal skull base surgery. J. Neurosurg..

[4] Kassam A.B., Prevedello D.M., Carrau R.L., Snyderman C.H., Thomas A., Gardner P., Zanation A., Duz B., Stefko S.T., Byers K. (2011). Endoscopic endonasal skull base surgery: Analysis of complications in the authors’ initial 800 patients. J. Neurosurg..

[5] Harvey R.J., Parmar P., Sacks R., Zanation A.M. (2012). Endoscopic skull base reconstruction of large dural defects: A systematic review of published evidence. Laryngoscope.

[6] Kessler R.A., Garzon-Muvdi T., Kim E., Ramanathan M., Lim M. (2019). Utilization of the nasoseptal flap for repair of cerebrospinal fluid leak after endoscopic endonasal approach for resection of pituitary tumors. Brain Tumor Res. Treat..

[7] Eloy J.A., Kuperan A.B., Choudhry O.J., Harirchian S., Liu J.K. (2012). Efficacy of the pedicled nasoseptal flap without cerebrospinal fluid (CSF) diversion for repair of skull base defects: Incidence of postoperative CSF leaks. Int. Forum Allergy Rhinol..

[8] Guo X., Zhu Y., Hong Y. (2020). Efficacy and safety of intraoperative lumbar drain in endoscopic skull base tumor resection: A meta-analysis. Front. Oncol..

[9] Soudry E., Turner J.H., Nayak J.V., Hwang P.H. (2014). Endoscopic reconstruction of surgically created skull base defects: A systematic review. Otolaryngol. Head Neck Surg..

[10] CRANIAL Consortium (2024). The impact of obesity on rates of post-operative CSF leak following endoscopic skull base surgery: Results from a prospective international multi-centre cohort study. Front. Endocrinol..

[11] Dlouhy B.J., Madhavan K., Clinger J.D., Reddy A., Dawson J.D., O’Brien E.K., Chang E., Graham S.M., Greenlee J.D.W. (2012). Elevated body mass index and risk of postoperative CSF leak following transsphenoidal surgery. J. Neurosurg..

[12] Senior B.A., Jafri K., Benninger M. (2001). Safety and efficacy of endoscopic repair of CSF leaks and encephaloceles: A survey of the members of the American Rhinologic Society. Am. J. Rhinol..

[13] Ahmed O.H., Marcus S., Tauber J.R., Wang B., Fang Y., Lebowitz R.A. (2017). Efficacy of Perioperative Lumbar Drainage following Endonasal Endoscopic Cerebrospinal Fluid Leak Repair. Otolaryngol. Head Neck Surg..

[14] Tien D.A., Stokken J.K., Recinos P.F., Woodard T.D., Sindwani R. (2016). Cerebrospinal fluid diversion in endoscopic skull base reconstruction: An evidence-based approach to the use of lumbar drains. Otolaryngol. Clin. N. Am..

[15] Stokken J., Recinos P.F., Woodard T., Sindwani R. (2015). The utility of lumbar drains in modern endoscopic skull base surgery. Curr. Opin. Otolaryngol. Head Neck Surg..

[16] Liu J.K., Schmidt R.F., Choudhry O.J., Shukla P.A., Eloy J.A. (2012). Surgical nuances for nasoseptal flap reconstruction of cranial base defects with high-flow cerebrospinal fluid leaks after endoscopic skull base surgery. Neurosurg. Focus..

[17] Zanation A.M., Carrau R.L., Snyderman C.H., Germanwala A.V., Gardner P.A., Prevedello D.M., Kassam A.B. (2009). Nasoseptal flap reconstruction of high flow intraoperative cerebral spinal fluid leaks during endoscopic skull base surgery. Am. J. Rhinol. Allergy.

[18] Xiong Y., Liu Y., Xin G., Xie S., Luo H., Xiao L., Wu X., Hong T., Tang B. (2022). Exploration of the causes of cerebrospinal fluid leakage after endoscopic endonasal surgery for sellar and suprasellar lesions and analysis of risk factors. Front Surg..

[19] Eloy J.A., Shukla P.A., Choudhry O.J., Singh R., Liu J.K. (2012). Assessment of frontal lobe sagging after endoscopic endonasal transcribriform resection of anterior skull base tumors: Is rigid structural reconstruction of the cranial base defect necessary?. Laryngoscope.

[20] Raper D., Komotar R., Starke R., Anand V., Schwartz T. (2011). Endoscopic versus open approaches to the skull base: A comprehensive literature review. Oper. Tech. Otolaryngol.-Head Neck Surg..

[21] Kono Y., Prevedello D.M., Snyderman C.H., Gardner P.A., Kassam A.B., Carrau R.L., Byers K.E. (2010). One thousand endoscopic skull base surgical procedures demystifying the infection potential: Incidence and description of postoperative meningitis and brain abscesses. Infect. Control Hosp. Epidemiol..

[22] Ivan M., Iorgulescu B., El-Sayed I., McDermott M., Parsa A., Pletcher S.D., Jahangiri A., Wagner J., Aghi M.K. (2015). Risk factors for postoperative cerebrospinal fluid leak and meningitis after expanded endoscopic endonasal surgery. J. Clin. Neurosci..

[23] Anji M., Mineharu Y., Kikuchi M., Nakagawa T., Sakamoto T., Yamashita M., Matsunaga M., Kuwata F., Kitada Y., Terada Y. (2022). Intraoperative Cerebrospinal Fluid Leak Graded by Esposito Grade Is a Predictor for Diabetes Insipidus After Endoscopic Endonasal Pituitary Adenoma Resection. World Neurosurg..

[24] Pang J.C., Liu D.H., Hong E.M., Frank M., Roman K.M., Jung J., Abiri A., Nguyen T.V., Bitner B.F., Hsu F.P.K. (2024). Predictors of length of postoperative stay following endoscopic skull base surgery with intraoperative CSF leak. J. Neurosurg..

